# Premarket Evidence and Postmarketing Requirements for Real-Time Oncology Review Indication Approvals

**DOI:** 10.1001/jamanetworkopen.2024.9233

**Published:** 2024-05-01

**Authors:** Maryam Mooghali, Ayman Mohammad, Joshua D. Wallach, Aaron P. Mitchell, Joseph S. Ross, Reshma Ramachandran

**Affiliations:** 1 Department of Internal Medicine, Section of General Internal Medicine, Yale School of Medicine, New Haven, Connecticut; 2 Yale Collaboration for Regulatory Rigor, Integrity and Transparency (CRRIT), Yale School of Medicine, New Haven, Connecticut; 3 Icahn School of Medicine at Mount Sinai, New York, New York; 4 Department of Epidemiology, Rollins School of Public Health, Emory University, Atlanta, Georgia; 5 Department of Epidemiology and Biostatistics, Memorial Sloan Kettering Cancer Center, New York, New York; 6 Department of Health Policy and Management, Yale School of Public Health, New Haven, Connecticut; 7 Center for Outcomes Research and Evaluation, Yale-New Haven Health System, New Haven, Connecticut

## Abstract

This cross-sectional study evaluates the use of the US Food and Drug Administration’s Real-Time Oncology Review (RTOR) program in confirming the effectiveness of cancer drugs.

## Introduction

The US Food and Drug Administration (FDA) launched the Real-Time Oncology Review (RTOR) program in 2018 to support earlier review of oncology therapeutics expected to be a substantial improvement over available therapy based on straightforward study designs and easily interpreted end points.^1^ Although RTOR applications have been associated with shorter median approval times,^2,3^ little is known about the evidence supporting RTOR indication approvals. Accordingly, we examined FDA indication approvals that underwent RTOR; characterized their supporting evidence; and established, when applicable, whether postmarketing studies were required to confirm clinical effectiveness.

## Methods

We used the Drugs@FDA database to identify all oncology indication approvals between February 1, 2018, and December 31, 2023. We then searched FDA approval packages, FDA announcements, and Google, in that order, to ascertain whether RTOR was used (eFigure in Supplement 1). In accordance with the Common Rule, this cross-sectional study was exempt from ethics review and informed consent because it used public nonidentifiable data. We followed the STROBE reporting guideline.

Using Drugs@FDA, we identified approval type (supplemental or original), first-in-class status,^4^ regulatory approval pathway (accelerated or traditional), efficacy end points (clinical or surrogate, per FDA definitions),^5^ sample size, and National Clinical Trial (NCT) numbers of pivotal trials supporting FDA approval. With NCT numbers, we searched ClinicalTrials.gov to extract trial design characteristics, including use of randomization, comparator group, and blinding, and start and primary completion dates. For indications approved based on only surrogate end points, we ascertained whether postmarketing studies were required to confirm clinical effectiveness.

Analyses were performed using Fisher exact and Mann-Whitney tests in RStudio, version 2023.09.1 + 494 (Posit Software). Two-tailed *P* < .05 was considered statistically significant.

## Results

The FDA approved 363 new oncology indications between 2018 and 2023, of which 76 (21%) underwent RTOR based on 84 pivotal trials. Of these 76 approvals, 22 (29%) were original and 54 (71%) were supplemental. Nine (41%) original approvals had a first-in-class status.

Among RTOR indication approvals, 15 (20%) received accelerated and 61 (80%) received traditional approvals (Table 1). All 15 accelerated approvals were based on pivotal trials using surrogate markers as primary end points and had FDA-required postmarketing studies confirming effectiveness. Among the 61 traditional approvals, 21 (34%) were based on pivotal trials using clinical outcomes for at least 1 primary end point and 7 (11%) used clinical outcomes for at least 1 secondary end point; 33 (54%) used only surrogate markers as primary and secondary end points. Among approvals using surrogate end points, 1 (3%) had required postmarketing studies confirming effectiveness as part of a safety study.

**Table 1.** Characteristics of the Food and Drug Administration Indication Approvals With Real-Time Oncology Review From 2018 to 2023

Characteristic	No. (%) (n = 76)		*P* value
	Traditional approval (n = 61)	Accelerated approval (n = 15)	
Indication approval type			
Supplemental	49 (80)	5 (33)	<.001
Original	12 (20)	10 (67)	
End point of pivotal trial			
At least 1 clinical primary end point	21 (34)	0	.008
No clinical primary end point but at least 1 clinical secondary end point	7 (11)	0	
Only surrogate markers as primary and secondary end points	33 (54)	15 (100)	
Required postmarketing studies for studies based on surrogate end points (n = 48)			
No	32 (97)	0	<.001
Yes	1 (3)	15 (100)	
Primary end point of postmarketing study (n = 15)^a^			
At least 1 clinical end point	NA	6 (40)	NA
Same surrogate end point used in the pivotal trial	NA	5 (33)	
Different surrogate end point	NA	4 (27)	

Abbreviations: NA, not available.

^a^ The postmarketing study for the Real-Time Oncology Review indication with traditional approval was not located; thus, we could not identify the corresponding primary end point.

Of the 84 pivotal trials, 16 (19%) supported accelerated and 68 (81%) supported traditional approvals (Table 2). Pivotal trials supporting accelerated approvals were less likely to include placebo or active comparators (4 [25%] vs 58 [85%]; *P* < .001), enrolled fewer patients (median [IQR], 108 [88-131] vs 612 [419-759]; *P* < .001), and took longer to complete (median [IQR], 69.4 [53.5-83.5] vs 40.1 [32.1-51.2] months; *P* = .01) than trials supporting traditional approvals.

**Table 2.** Characteristics of Pivotal Trials Supporting Approvals of Indications With Real-Time Oncology Review From 2018 to 2023

Characteristic	No. (%) (n = 84)		*P* value
	Traditional approval (n = 68)	Accelerated approval (n = 16)	
Study type			
Interventional	68 (100)	15 (94)	.21
Observational	0	1 (6)	
Sample size, median (IQR)	612 (419-759)	108 (88-131)	<.001
Study duration, median (IQR), mo	40.1 (32.1-51.2)	69.4 (53.5-83.5)	.01
Primary efficacy end point			
Clinical outcome	21 (31)	0	.01
Surrogate marker	47 (69)	16 (100)	
Study design			
Single arm with no comparator	10 (15)	12 (75)	<.001
Multiple arm	58 (85)	4 (25)	
Comparator			
Placebo	18 (31)	1 (25)	.80
Active comparator	40 (69)	3 (75)	
Allocation			
Randomized	58 (100)	4 (100)	>.99
Nonrandomized	0	0	
Blinding			
Double-, triple-, or quadruple-blinded	20 (34)	1 (25)	.52
Single-blinded (outcome assessor)	4 (7)	0	
Open label	34 (59)	3 (75)	

## Discussion

One-fifth of new FDA oncology indication approvals were reviewed under the RTOR program since its 2018 inception. These approvals, including those under the traditional pathway, were often supported by pivotal trials using surrogate end points. The RTOR indications with traditional approvals based on surrogate end points rarely had postmarketing requirements to confirm their clinical benefit. Outside of accelerated approvals, the FDA could require additional postmarketing studies to ensure flexible RTOR approvals based on surrogate end points.^6^

Study limitations include the inability to account for non–publicly available information, including the total number of RTOR submissions and factors weighed by the FDA when evaluating benefits and risks. While RTOR is increasingly used to support earlier approvals for oncology indications, these approvals were based on pivotal trials with less robust designs, use of surrogate primary end points, and no requirements to confirm clinical benefit, potentially contributing to clinical uncertainty among patients and clinicians.

## Notes

Supplement 1. **eFigure.** Sample Identification

Supplement 2.
Data Sharing Statement
