# Supplementary material for: Premarket Evidence and Postmarketing Requirements for Real-Time Oncology Review Indication Approvals
Source: JAMA Netw Open. 2024 May 1;7(5):e249233. doi: 10.1001/jamanetworkopen.2024.9233 (PMC11063797; doi:10.1001/jamanetworkopen.2024.9233)
Supplement: Supplement 1. — eFigure. Sample Identification [file jamanetwopen-e249233-s001.pdf]

Supplemental Online Content

Mooghali M, Mohammad A, Wallach JD, Mitchell AP, Ross JS, Ramachandran R. Premarket evidence and postmarketing requirements for Real-Time Oncology Review indication approvals. *JAMA Netw Open*. 2024;7(5):e249233.
doi:10.1001/jamanetworkopen.2024.9233

eFigure. Sample Identification

This supplemental material has been provided by the authors to give readers additional information about their work.

© 2024 Mooghali M et al. *JAMA Network Open*

eFigure. Sample Identification

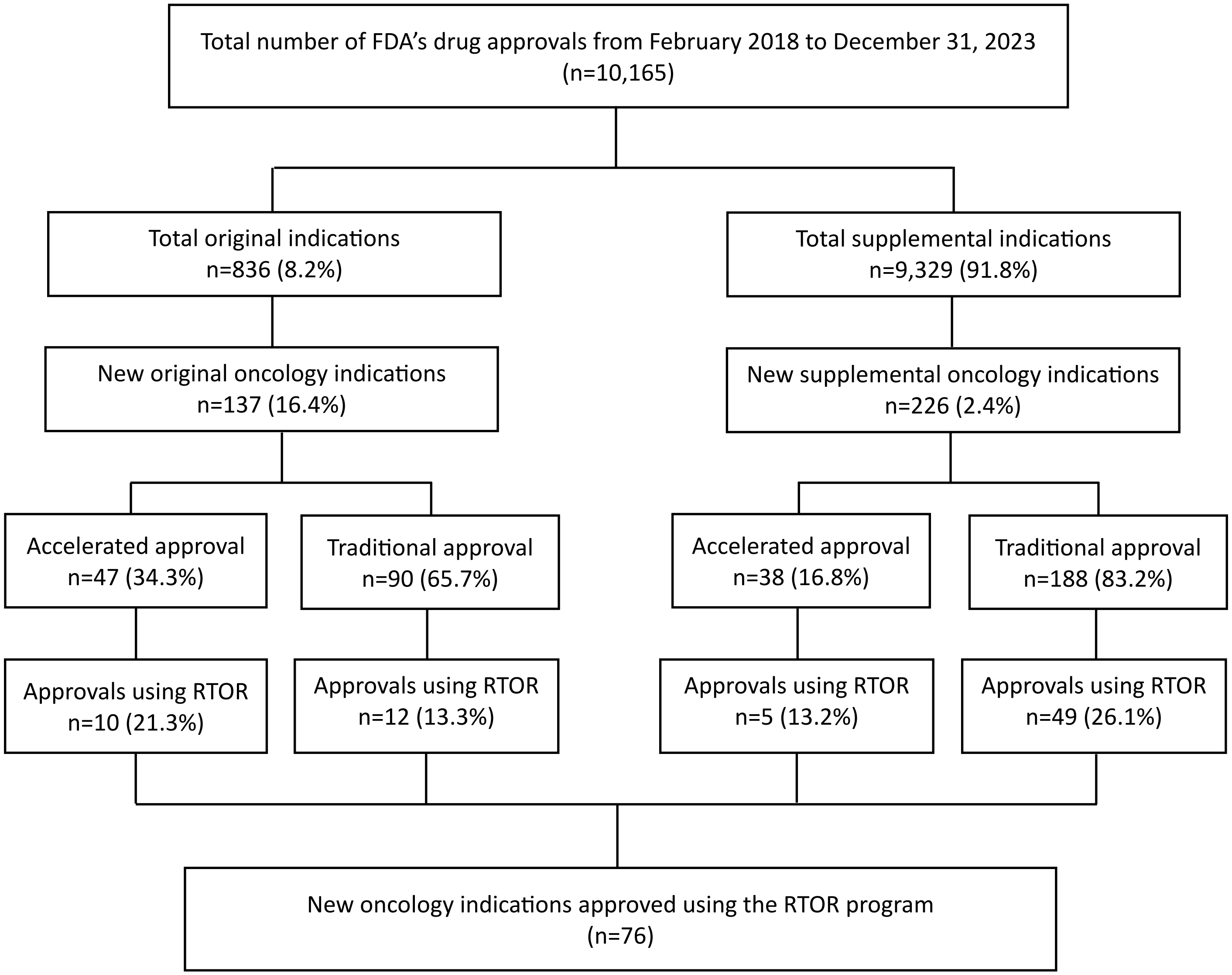

© 2024 Mooghali M et al. *JAMA Network Open*
