# Supplementary material for: Premarket Evidence and Postmarketing Requirements for Real-Time Oncology Review Indication Approvals
Source: JAMA Netw Open. 2024 May 1;7(5):e249233. doi: 10.1001/jamanetworkopen.2024.9233 (PMC11063797; doi:10.1001/jamanetworkopen.2024.9233)
Supplement: Supplement 2. — Data Sharing Statement [file jamanetwopen-e249233-s002.pdf]

Data Sharing Statement

Mooghali. Premarket Evidence and Postmarketing Requirements for Real-Time Oncology Review Indication Approvals. *JAMA Netw Open*. Published May 01, 2024.
doi:10.1001/jamanetworkopen.2024.9233

Data
Data available: No

Additional Information
Explanation for why data not available: Relevant data are available on reasonable request from the corresponding author.
